# Analyzing key constraints to biogas production from crop residues and manure in the EU—A spatially explicit model

**DOI:** 10.1371/journal.pone.0171001

**Published:** 2017-01-31

**Authors:** Rasmus Einarsson, U. Martin Persson

**Affiliations:** Division of Physical Resource Theory, Department of Energy and Environment, Chalmers University of Technology, Gothenburg, Sweden; University of Vermont, UNITED STATES

## Abstract

This paper presents a spatially explicit method for making regional estimates of the potential for biogas production from crop residues and manure, accounting for key technical, biochemical, environmental and economic constraints. Methods for making such estimates are important as biofuels from agricultural residues are receiving increasing policy support from the EU and major biogas producers, such as Germany and Italy, in response to concerns over unintended negative environmental and social impacts of conventional biofuels. This analysis comprises a spatially explicit estimate of crop residue and manure production for the EU at 250 m resolution, and a biogas production model accounting for local constraints such as the sustainable removal of residues, transportation of substrates, and the substrates’ biochemical suitability for anaerobic digestion. In our base scenario, the EU biogas production potential from crop residues and manure is about 0.7 EJ/year, nearly double the current EU production of biogas from agricultural substrates, most of which does not come from residues or manure. An extensive sensitivity analysis of the model shows that the potential could easily be 50% higher or lower, depending on the stringency of economic, technical and biochemical constraints. We find that the potential is particularly sensitive to constraints on the substrate mixtures’ carbon-to-nitrogen ratio and dry matter concentration. Hence, the potential to produce biogas from crop residues and manure in the EU depends to large extent on the possibility to overcome the challenges associated with these substrates, either by complementing them with suitable co-substrates (e.g. household waste and energy crops), or through further development of biogas technology (e.g. pretreatment of substrates and recirculation of effluent).

## Introduction

In order to reduce emissions of greenhouse gases and limit its contribution to global climate change, the Renewable Energy Directive (RED) of the European Union (EU) requires member states to source at least 10% of transport fuels from renewable sources by 2020. Achieving this target sustainably, without unintended negative consequences, is a challenge. So far, increased demand for renewable transportation fuels has primarily been met by so-called conventional biofuels, produced from food crops (e.g., wheat ethanol and rapeseed biodiesel). However, the rapid increase in conventional biofuel consumption has caused concerns over the impacts increased biofuel feedstock demand has on agricultural commodity markets, e.g., raising food prices [1], and causing indirect land-use change that weakens or reverses the climate gains from biofuel use [2].

The European Commission has responded to these concerns by proposing amendments to the RED [3], mandating that biofuels counted towards the RED target achieve at least 60% greenhouse gas savings, limiting the share of conventional biofuels (from food crops on agricultural land) to 7% of total transport energy demand [4], and incentivizing the production of advanced biofuels produced from feedstocks that do not compete for land with food and feed production.

One example of the latter is biogas produced from municipal and industrial wastes or agricultural residues through anaerobic fermentation and upgraded to vehicle fuel quality biogas. (Biogas produced through anaerobic fermentation commonly consists of 50–75% methane (CH_4_) and 25–50% carbon dioxide (CO_2_). To be used as a transportation fuel, or to be injected in the natural gas grid, the biogas needs to be dried, desulfurized and removed of CO_2_, raising the CH_4_-content above approximately 96%.)

In 2013 it is estimated that there were nearly 14,000 anaerobic digesters in the EU, producing a total of 560 PJ of biogas [5]. Still, much of the biogas produced in the EU is currently produced from dedicated energy crops. In Germany—hosting over half of all EU biomethane plants—three quarters of substrates are dedicated energy crops (primarily maize silage). Consequently, the German government, facing the same concerns over land competition, food price increases and indirect land-use change as from conventional biofuels, recently removed subsidies for biogas produced from energy crops to instead encourage the use of agricultural waste [5]. A similar development is taking place in the third largest biogas producer country in the EU, Italy. The EU Commission has also highlighted the need for improving the greenhouse gas performance of biogas and biomethane production by raising the share of agricultural waste, manure and slurry as feedstocks [6].

Given these policy developments, both on EU level and in individual member states, pertinent questions are: (1) How large is the potential for producing biogas from agricultural residues, such as straw and manure, in the EU, (2) how is this potential distributed across member states, and (3) what are the main limiting factors for this potential? These are the questions we set out to answer in this paper.

Several studies have already estimated the availability of agricultural waste for bioenergy in the EU (see [7] and references therein), with most studies arriving at estimates of around 1–2 EJ/year (though results vary widely due to different assumptions regarding technical and environmental constraints). However, these top-down assessments stop at the potential availability of residues, and do not account for the technical and economic constraints involved in converting substrates to biogas.

A number of bottom-up studies, on the other hand, have done detailed analyses of biogas potential from agricultural residues at local to regional scale, accounting for a wide array of technical, economic and environmental constraints (see, e.g., [8–12], as well as [13] for an application outside the EU). However, because of their detailed nature, it would be nearly impossible to scale up these studies to the EU level. Also, most of these studies do not systematically explore how the estimated potentials are affected by changes in the imposed constraints.

Lacking in the current literature, therefore, is an EU-level analysis of the potential for biogas production from crop residues and manure, which accounts for how key technical, economic and environmental constraints affect both the absolute magnitude and spatial distribution of the potential. It is important to analyze how the stringency of these constraints affects the biogas potential since the constraints are bound to change over time, due to changes in technology (e.g., further development of processes for dry fermentation), economic factors (e.g., fuel price changes affecting the economically viable collection radius for substrates), and policies (e.g., changes in subsidies favoring small-scale biogas plants, as is currently happening in Germany and Italy).

Here we present a new method developed to analyze the potential for biogas production from crop residues and manure in the EU. The model provides spatially explicit biogas potentials with a resolution of 250 m, by downscaling agricultural statistics on crop production (akin to the method proposed in [14]) and livestock populations. We represent key technical, economic and environmental constraints by a limited number of model parameters (maximum substrate removal rate and transportation distance, minimum viable plant size, and minimum/maximum carbon-to-nitrogen (C:N) ratio and dry matter (DM) content of substrate mix), allowing us to explicitly model how changes in these parameters affect the estimated potential in different parts of the EU.

The rest of this paper is structured as follows. In the Methods section, we first describe how the regional availability of manure and crop residues are estimated and spatially downscaled. The section ends with a description of how technical, biochemical and economic constraints are represented in the biogas production model. In the Results section we present the geographical distributions of substrates and the biogas potential, and describe how the biogas potential changes depending on the stringency of the constraints. We end by discussing the usefulness of our method, the relative importance of different constraints and what conclusions can be drawn for future research on the limitations to biogas production from crop residues and manure.

## Materials and Methods

**Fig 1:**
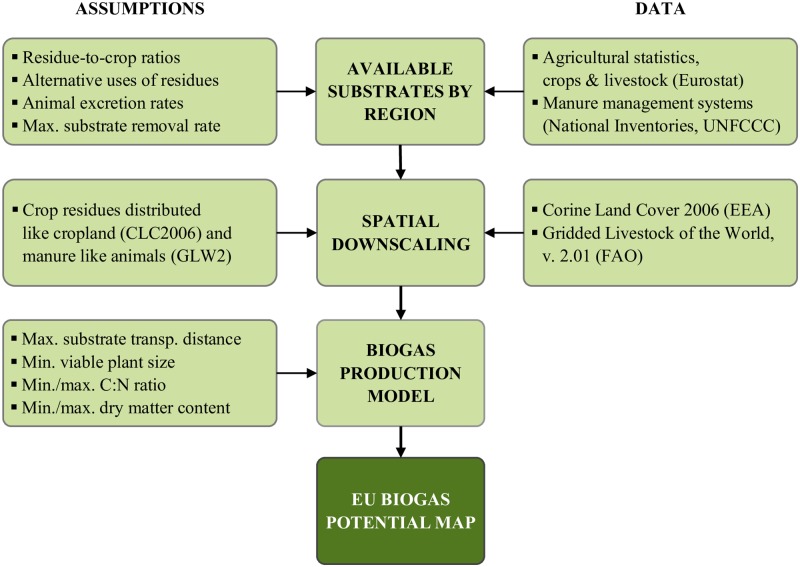
Illustration of the method and data sources used. First, available substrate amounts were estimated using subnational statistics. Second, the substrate amounts were spatially downscaled using land cover and livestock population datasets. Third, economic and technical limitations were analyzed using a model of a biogas plant. The local production potentials were upscaled to the EU level to obtain an overall potential estimate.

Before making a detailed description of the method and the data sets used, we briefly summarize the three steps of the analysis (see also Fig 1).

First, the amounts of manure and crop residues available for biogas production in the EU were estimated, using subnational agricultural statistics and several other data sources which are further discussed below. The result of this was an estimate of the annually available substrates, on the same statistical aggregation level as the agricultural statistics used (Eurostat NUTS2).

Second, the regional substrate amounts were spatially downscaled to a much finer resolution using a land cover map, Corine Land Cover [15], and a livestock population density map, Gridded Livestock of the World 2, [16]. Downscaling of the substrate densities enables estimation of the mix of substrates that can be collected at any given location.

Third and last, the biogas potential was estimated by maximizing the use of available substrates in each geographical location, taking into account technical and economic constraints such as the minimum viable plant size, reasonable collection distances for different substrates, and the DM concentration and C:N ratio of substrates. This local potential measure was then upscaled to the EU level to obtain an overall potential estimate.

### Regional estimation of substrate availability

#### Crop production

The management of crop residues is highly variable depending on, e.g., competing uses, weather and climate conditions, soil properties, and crop rotations. Some residues, primarily straw, are directly used elsewhere, e.g. as animal bedding material, in mushroom production, or incinerated for energy, but a large fraction of crop residues is also left in the fields. In general, there is little data available on the produced quantities of crop residues and how they are managed, but there have been some efforts to estimate how much could be used for energy purposes using national statistics [17] and subnational statistics [14]. Our method for estimating the supply of crop residues is similar to these methods, but extended with new data.

The production of collectable residues was estimated for wheat, barley, rye, rapeseed and turnip rape, grain maize, sugar beets, and sunflower. The production of residues was obtained by multiplying the crop harvests by residue-to-crop ratios. However, all the produced residues cannot be sustainably removed from the fields if soil organic matter is to be preserved. This constraint is further discussed in a separate section below.

Subnational agricultural production statistics are published by Eurostat according to the NUTS classification (Nomenclature of territorial units for statistics). The 28 member states of EU constitute the NUTS0 level which is further subdivided in about 130 regions on NUTS1 level and about 300 regions on NUTS2 level. For many crops in most countries, harvested amounts are published by Eurostat on NUTS2 level each year. Where such harvest data were missing, we instead estimated harvests on NUTS2 level by multiplying national-level yields by NUTS2 level data on planted areas from the detailed triannual Eurostat farm structure survey. Throughout the calculations, we used averages over the years 2009–2011 where available. See the S1 Appendix for further details.

The crop harvests were multiplied by residue-to-crop ratios indicating how much residues are produced per unit of harvest (see Table 1). The residue-to-crop ratios were taken from Swedish measurements on wheat, barley, oats, rye, rapeseed [18] and sugar beet [19], and from the review presented in [17]. Although the Swedish measurements of residue-to-crop ratios are generally in line with the values collected in [17], it should be noted that sources sometimes disagree by 50% or more, likely depending both on differences in measurement methods and on variation across countries, crop varieties, and years. We prioritized the Swedish measurements [18, 19] since they are detailed, clearly described, internally consistent, and relatively recent.

**Table 1:** Residue-to-crop production ratios, the assumed quotients between total (wet) weight of crop residues and reported crop production (kernel/seed weight).

Crop	Residue-to-crop ratio	Source
Wheat	0.9	[18]
Rye	1.1	[18]
Barley	0.7	[18]
Oats	0.8	[18]
Rapeseed and turnip rape	1.2	[18]
Maize	1	[17]
Sugar beets	0.6	[19]
Sunflower	2	[17]

#### Sustainable removal rate of residues

There are at least two reasons why only a part of the produced crop residues can be sustainably utilized. First, crop residues are an important source of soil organic matter and therefore not all residues can be removed from fields without creating unacceptable impacts on soil quality. Second, if the weather is very wet after harvest, the quality of the residues may be degraded and there may be severe soil compaction if heavy machinery is used for removal of residues. These are both serious concerns which farmers legitimately may consider before utilizing or selling their crop residues.

The sources reviewed in [17] report sustainable removal rates mostly in the range 30–60% for the crops considered here, but it should be noted that both higher and lower values could apply in some locations, depending on soil type, climate, topography, other sources of soil organic matter, etc. This geographical variation in sustainable removal rate was estimated in [20] using results from a pan-European assessment of topsoil organic carbon [21] based on the CENTURY model. It is also likely that higher removal rates are sustainable in a biogas production system if the digestate is returned to the same fields. However, even if all the digestate is returned, the biogas production unavoidably entails a considerable amount of carbon removal.

We used the simple approach to use a single maximum residue removal rate throughout Europe for all crop residues. Following [17], we chose the maximum removal rate of 40% in the base scenario, and to complete the picture we present an extensive sensitivity analysis of this parameter in the Results section.

#### Alternative uses of crop residues

The most important alternative use for crop residues is straw as bedding for animals. We estimated the amount of straw used for bedding as explained in the section on manure below and then computed the straw balance separately in each NUTS2 region by subtracting the bedding straw from the available resource, based on the assumption that straw for bedding is not moved between regions. In a few regions where the estimated demand for bedding exceeded the amount of available straw we limited the bedding use to equal the available amount.

As previously mentioned, some straw is also used for mushroom cultivation. However, we chose to omit this term since it is typically quite small (less than 1% of the total production [17]) and because reliable data are lacking. Other energy uses (primarily incineration of straw) were assumed to be zero since the aim was to study an alternative way of using crop residues and manure for energy. All other uses of residues, e.g. in industrial applications, were approximated to zero since they are likely very marginal, around 1% of straw production [17].

#### Manure substrates

Manure production was estimated for dairy cows and other cattle, breeding and fattening pigs, broilers and laying hens, divided into different manure management systems. The calculation was based on animal population statistics from Eurostat on the finest level publicly available (NUTS1 for Germany, NUTS2 for all other countries in EU28). The S1 Appendix contains further details on data sources.

In principle, it is straightforward to estimate the available manure resources by multiplying each animal population by its average excretion rate and the shares excreted in different manure management systems. However, neither of the latter two parameters are systematically and comparably measured across the EU. Therefore we must rely on incomplete survey data and expert judgement for these parameters.

We chose to use a single excretion rate for each animal class across the EU, see Table 2. However, in reality the excretion rates vary depending on animal weight, feed characteristics, growth rate, milk yield of cows, etc. Since there is substantial variation in these parameters between EU countries there are likely also differences in average excretion rates.

**Table 2:** Assumed excretion and total manure production per animal head for different animals and manure management systems. Excretion values for cattle and pigs from [27]. Excretion for poultry based on [28]. See the S1 Appendix for details on manure management and bedding.

Animal	Excretion (kg VS head^-1^ d^-1^)	System	Manure incl. bedding (kg VS head^-1^ d^-1^)
Dairy cows	5.1	liquid	5.1
		solid	10.2
Other cattle	2.6	liquid	2.6
		solid	5.2
Breeding pigs	0.5	liquid	0.5
		solid	1.0
Fattening pigs	0.3	liquid	0.3
		solid	0.6
Laying hens	8 ⋅ 10^−3^	liquid	8 ⋅ 10^−3^
		solid	8 ⋅ 10^−3^
Broilers	5 ⋅ 10^−3^	liquid	5 ⋅ 10^−3^
		solid	5 ⋅ 10^−3^

One way to account for the variation between countries would be to use the excretion rates reported in the National Inventory Reports (NIRs) to the UNFCCC. In some cases these numbers may be more accurate, especially where country-specific models incorporating detailed data are used. However, it is hard to judge how the overall accuracy of the calculation would be affected given the large variations in methods used by different countries.

Another method for predicting excretion rates is to use feed digestion models for the different animals (see e.g. [22, 23]), but such models typically require detailed data on both animals and feed characteristics which are also not systematically collected across the EU. Despite these difficulties, this approach has been taken e.g. in [24] to calculate manure-related greenhouse gas emissions using the CAPRI model with estimated animal feed data.

In summary, adding more detailed estimates of excretion rates is a possible extension of the method presented here. Compared to detailed model systems such as CAPRI, the approach taken here improves model transparency and ease of sensitivity analysis at the expense of potentially reduced accuracy.

#### Manure management

Manure management systems were included in the model for two distinct reasons: (1) because some of the excretions fall on pastures and we assumed that this portion cannot be collected, and (2) because manure is often mixed with significant amounts of cereal straw or other bedding materials. This addition of dry materials into solid manure is important to consider because it changes the characteristics of the manure as a biogas substrate by increasing its DM content and C:N ratio, and affects the techniques and costs associated with transportation. Furthermore, straw bedding reduces the amount of straw available elsewhere.

Information sources on manure management practices in the EU are scarce, typically based on expert judgement rather than measurements, and either qualitative or highly uncertain. It is widely recognized that manure management practices differ significantly both between and within countries. Furthermore, comparison of different information sources is complicated since no standardized terminology is agreed upon. Some recent surveys covering all or most of the EU are found in [24–26] and references therein.

In face of this data scarcity, we chose to make a simple quantitative description of manure management systems which can be expected to capture the most important variations across the EU. Three management systems were included and assumed to be identical for our purposes wherever they are used: liquid manure, solid manure, and pasture. Country-specific data on the shares of manure in these different management systems for different livestock classes (dairy cows, other cattle, breeding swine, market swine, hens, and broilers) were taken from the NIRs submitted by EU countries. We used average values over the years 2009–2011 where available. (See the S1 Appendix for details on our manure management model and the data sources used.)

For liquid manure, we assumed that no bedding material is used. For solid manure, we assumed a single bedding material (straw for cattle and pigs, wood shavings for poultry) added in fixed proportions. In a few NUTS2/NUTS1 regions, this estimated demand for bedding straw exceeded the collectable resource, indicating that we had overestimated the straw use and/or underestimated the supply. In these cases, we assumed that no straw is transported across region boundaries and instead changed shares of straw-based solid manure into liquid manure until the straw use was sufficiently small. Manure management was adjusted only in the NUTS1/NUTS2 regions where needed.

The resulting production of manure-based biogas substrates per animal head in different manure management systems is listed in Table 2. For each animal species and manure management system, assumed compositions (concentrations of volatile solids (VS), DM, C, and N) are listed in Table 3. Different sources sometimes disagree considerably regarding the chemical composition of manure, which is likely a reflection of corresponding variation in animal feed composition and in manure management systems, e.g. in the rate of straw and water addition, or presence of urine separation in animal houses. In conclusion, the estimates of manure substrates presented here are far from perfect, but it is almost impossible to do better with the currently available data.

**Table 3:** Properties of substrates. Dry matter (DM) expressed as fraction of total weight. Volatile solids (VS) expressed as fraction of DM, and carbon (C) and nitrogen (N) expressed as fractions of VS. For conversion of methane volume to higher heating value (HHV) the factor 40 MJ m^−3^ was used. Compositions are based on data reported in [19, 26, 29–34]. Methane yields based on data from [19, 35–38].

		DM %	VS/DM %	C/VS %	N/VS %	Methane yield (m^3^ CH_4_ / Mg VS)
Cattle manure	liquid	8	80	55	7	200
	solid	20	85	′′	3.5	′′
Pig manure	liquid	6	80	′′	10	′′
	solid	20	85	′′	5	′′
Chicken manure	liquid	30	70	′′	9	250
	solid	70	70	′′	9	250
Crop residues	straw	85	90	′′	0.5	200
	maize	′′	′′	′′	′′	′′
	sunflower	′′	′′	′′	′′	′′
	sugar beet	13	′′	′′	2.5	300

### Spatial downscaling of substrate amounts

The following two sections describe our method for spatially downscaling the substrate amounts; in other words, how the subnational regional estimates were combined with geospatial datasets to produce spatially explicit density maps of all the substrates in resolution fine enough to model the production potential of biogas plants.

#### Downscaling of crop residue availability

To estimate the spatial distribution of crop residue production we used the Corine Land Cover 2006 (CLC2006) dataset, version 17 [15], a land cover map classifying land and water bodies in 44 different classes based on computer-aided interpretation of satellite images. CLC2006 version 17 includes all EU28 countries except Greece. We used a raster version of CLC2006 with 250 m resolution.

The spatial distribution of crop production was estimated assuming that the above-mentioned crops are allocated to five land cover classes, weighted by the share of each land cover class likely to be cropland (see [39] for details). The classes and their assigned weights are as follows. Non-irrigated arable land: 100%; Permanently irrigated land: 100%; Annual crops associated with permanent crops: 50%; Complex cultivation patterns: 50%; and Land principally occupied by agriculture with significant areas of natural vegetation: 50%.

#### Downscaling of manure availability

The spatial downscaling of manure production was done in a similar manner as for crop production, but using the FAO Gridded Livestock of the World dataset, version 2.01 (GLW2) [16]. The GLW2 dataset is an estimation of animal population densities in a resolution of roughly 1 km, based on subnational animal population statistics and a statistical model with predictor variables describing vegetation, climate, topography and demography.

The estimated manure amounts in each region were distributed proportionally to the corresponding animal populations in GLW2. Note that the GLW2 animal classes (e.g. cattle and chickens) are more aggregated than the excretion categories (e.g. dairy cows and other cattle; broilers and laying hens). In other words, the difference in spatial distribution, e.g. between dairy cows and other cattle, is only accounted for down to the resolution of Eurostat animal statistics. At finer resolution, the GLW2 dataset used does not distinguish between dairy cows and other cattle, between broilers and laying hens, or between breeding and fattening pigs.

### Technical and economic limitations to biogas potential

The steps described above produced an estimate of how much manure and crop residues could theoretically be used as substrates in biogas production at a spatial scale of 250 m (by interpolating the GLW2 dataset to the same resolution as CLC2006). The next step was to estimate the potential for producing biogas from these substrates when placing a hypothetical biogas plant somewhere in the EU. In this estimate, we accounted for the following constraints:

- Maximum transportation distances for substrates
- Minimum viable plant size
- Minimum and maximum DM content
- Minimum and maximum C:N ratio
- The methane yield of different substrates

The following sections provide some background on these constraints before describing how they were modeled.

#### Transportation distances and minimum viable plant size

Larger biogas plant sizes are typically more cost-effective given a sufficient substrate supply. When the biogas is used to produce electricity, calculations indicate considerable returns to scale, perhaps a 30% cost reduction going from 150 to 1000 kW methane production (higher heating value, HHV) [40, 41].

In the base scenario, we chose a minimum plant size of 1 MW HHV, which is a typical size for power production. In alternative scenarios, we also present results for both higher and lower limits on the minimum production unit size. Requiring larger plant sizes is relevant especially if the gas is to be upgraded to vehicle fuel quality,. For biogas upgrading, the typical production scale is almost an order of magnitude larger than for power production: 1 MW HHV is a relatively small upgrading unit and many commercial units handle 5 MW HHV or more [42, 43].

However, larger plant sizes require longer transportation. In areas where substrate density is low, the collection of substrates and spreading of digestate may require uneconomically long transportation distances. An absolute upper limit for transport should be when the net energy balance of the operations turns negative, i.e. when more energy is used than the produced biogas output. For manure and straw this energetic break-even transportation distance can be some 200 km [44], but the economically feasible distance is much less. In the base scenario, a collection distance of 15 km for all substrates was assumed, in line with scenarios considered in [11, 44].

Of course, several other conditions apart from collection distances and production scale affect the economics of biogas production. The profitability is, for example, influenced by how the gas is utilized (power or biofuel), what the selling price is, whether there is economic support (e.g. feed-in tariffs), and what the cost of capital is. However, it is a daunting task to explicitly and consistently analyze all these parameters across the whole EU since they vary both over time and among member states.

As a first approximation, we therefore chose to only require a maximum collection radius and minimum plant size as described above. Since there are already thousands of commercial production plants operating under constraints similar to those assumed here, we presumed that more plants could be established in other locations, given costs, support systems, and energy prices not too unlike the present.

#### Dry matter content

Biogas processes can be divided into two main categories: wet and dry fermentation. The DM concentrations in reactors are typically around 10% for wet fermentation and in the range 15–35% for dry fermentation [45] (note that the DM concentration can be somewhat higher for the substrate mixture feeding the process since DM is continuously decomposed and removed). A number of chemical and physical substrate characteristics can influence which reactor type, mixing equipment, etc., are most suitable. For an introduction to this rather complicated topic, see e.g. [45] and references therein. Here it will suffice to note that DM content of the substrate mixture is one possible predictor of technical feasibility.

We chose to model wet digestion in the base scenario since this is the most commonly used technology and considerably more mature than dry fermentation. Still, it is worth to mention two technical options for lowering the DM concentration: (1) to dilute the substrates with fresh water and (2) to recirculate liquids from the reactor effluents. Neither of these methods are necessarily simple fixes. Dilution of very dry substrates may require very substantial amounts of fresh water. Recirculation can help lower the DM concentration and increase the methane yields, but it also causes accumulation of both organic and inorganic substances in the reactor which may inhibit the digestion process [46, 47]. To our knowledge, there are no examples of large-scale wet digesters currently using recirculation or fresh water dilution to deal with high proportions of crop residues.

There is also an economic aspect to the DM concentration, or rather the C concentration. In biogas plants using very dilute substrates such as pig slurry, co-substrates increasing the average C content can improve economic viability by increasing production per unit reactor volume [48] and decreasing costs for transportation of substrates and digestate. Therefore, a minimum DM concentration can be used as a rough proxy of the economic limits to using very water-rich substrate mixtures.

In the base scenario, maximum and minimum DM concentrations of 12% and 0% were assumed. For the reasons outlined above, both parameters were tested with a wide range of higher values in alternative scenarios.

#### C:N ratio and methane yields

Anaerobic digestion requires a balance between C and N content. The C:N ratio is an often-mentioned parameter because ammonia inhibition [49] is a potential obstacle to digestion of manure slurries with low C:N ratios. Very high C:N ratios should also be avoided since reactor stability and methane yield may decrease if N available for microbial growth is lacking.

Recent experiments on mixtures of dairy manure, chicken manure and rice straw [36] indicate optimal C:N ratios in the range 25–35. In contrast, a C:N ratio below 20 has been mentioned as state-of-the-art in Danish biogas plants [31], and optimal digestion results of leather fleshing waste and municipal solid waste mixtures have been found at C:N ratio of 15 [50]. We find this variation in reported optimal C:N ratios unsurprising, considering the complexity of digestion processes and the many possible confounding variables such as pH, temperature, other nutrient composition, etc.

In the base scenario, we chose a relatively wide range for possible C:N ratios for substrates, requiring it to be in the range 10–35.

The methane yield of substrates depends on their physical and biochemical properties and on digestion process parameters such as temperature and retention time. The physical and biochemical properties of substrates can also be changed through pretreatment, for example steam explosion, grinding or extrusion, or biological pretreatment. Pretreatment is particularly useful for some lignocellulosic substrates such as straw, which may otherwise be hard to digest [37, 51]. We assumed rather conservative methane yields (see Table 3), and it should be noted that they could be substantially higher with the right combination of pretreatment, substrate mixtures and microbial properties in digesters.

#### Local potential estimation using a stylized biogas plant model

After estimating the regional substrate amounts and spatially downscaling them, two more steps were taken to assess the limitations to biogas production from manure and crop residues. First, a stylized model of a biogas plant was introduced to derive a location-specific measure of the biogas potential, given the constraints discussed in the previous sections. Second, a weighted average of this measure over the whole EU was used as an estimate of the overall potential and its limitations.

Consider a biogas plant placed in a point **p**. The available quantity *S*_*i*_ of each substrate was here computed as the integral of the substrate density over an area with radius *r*_*i*_ centered at **p** (in the area-preserving projection EPSG:3035, recommended by EEA for spatial analysis in the EU). The radius *r*_*i*_ associated with each substrate is a simplified representation of its economically feasible collection distance, which may depend e.g. on its energy density and the technology used for transportation. Since roads and topography were not included in the model, no adjustment was made for the very variable density, layout and quality of road networks.

As explained above, some of the available substrate quantities *S*_*i*_ may be impossible to utilize because the overall mixture is too dry or too wet, or has too high or too low C:N ratio, or if the substrates within the collection area are not enough to support the minimal plant size. The production potential is therefore obtained by maximizing production from all the available substrates, subject to these constraints.

For simplicity, we assumed that each substrate has a methane yield which is not affected by co-substrates, or in other words that there are no synergy effects of substrate mixtures. With this assumption, the biogas production is a linear function of the used substrate quantities, allowing us to formulate the production maximization as a linear optimization problem as follows:

$$\begin{array}{ll} \text{maximize} & \sum_{i}Y_{i}s_{i}\\ \text{subject to} & \\ & 0\leq s_{i}\leq S_{i}\;\forall i,\\ & D_{\text{min}}\leq \frac{\sum_{i}s_{i}}{\sum_{i}s_{i}/D_{i}}\leq D_{\text{max}},\\ & {\text{CN}}_{\text{min}}\leq \frac{\sum_{i}C_{i}s_{i}}{\sum_{i}N_{i}s_{i}}\leq {\text{CN}}_{\text{max}},\\ & P_{\text{min}}\leq \underset{i}{\sum }Y_{i}s_{i}. \end{array}$$

Here, the available substrate quantities at the point **p**, after accounting for other uses and the maximum crop residue removal rate, are denoted *S*_*i*_, and the utilized quantities are denoted *s*_*i*_. The quantities are measured as DM mass per unit time. The energy yield of each substrate is *Y*_*i*_ (energy/DM) so that the total energy output is *P* = ∑_*i*_ *s*_*i*_ *Y*_*i*_. The DM fraction (of the total weight) of each substrate is *D*_*i*_ and the corresponding limits on average DM concentration in the reactor infeed are *D*_min_ and *D*_max_. The C and N fractions are *C*_*i*_ and *N*_*i*_, and the limits on C:N ratios are denoted *CN*_min_ and *D*_max_. Finally, *P*_min_ is the minimum output effect of a plant.

There are two possible outcomes of this formulation: Either (1) the problem can be solved and the substrate flows *s*_*i*_ can be used to produce the effect *P* = ∑_*i*_ *Y*_*i*_ *s*_*i*_, or (2) the problem is infeasible (has no solutions), indicating that the available substrates cannot satisfy all the constraints. We interpreted infeasibility as *P* = 0.

As a benchmark for the production potential, we used the theoretical biogas production obtained if all the available substrates could be digested, i.e. *P*_max_ = ∑_*i*_ *Y*_*i*_ *S*_*i*_. The quantity *P*(**p**)/*P*_max_(**p**) is therefore a number between 0 and 1, equal to the utilized share of the theoretical potential at a point **p**.

Assumed values for the constraint parameters are listed in Table 4.

**Table 4:** Parameters for the constraints as assumed in the base scenario. Alternative scenarios were defined by varying two of the constraint parameters at a time, while keeping the others equal to the base scenario values.

Parameter	Symbol	Min	Max
DM concentration	*D*	0	12%
C:N ratio	CN	10	35
Plant size	*P*	1 MW (HHV)	–
Collection radius	*r*	–	15 km
Residue removal rate	*R*	–	40%

#### Upscaling of biogas potential to a larger area

Finally, the overall potential *P*_tot_ in some area *A*, was estimated as

$$\begin{array}{c} P_{\text{tot}}\left(A\right)=P_{\text{theoretical}}\left(A\right)\alpha \left(A\right), \end{array}$$

where *P*_theoretical_ is the theoretically maximal biogas production obtained assuming that all available substrates in the whole area can be digested. It is multiplied by a number *α*(*A*) (between zero and one), defined as the area’s average of the utilizable share in each point, *P*(**p**)/*P*_max_(**p**), weighted by the maximum potential, i.e.

$$\begin{array}{c} \alpha \left(A\right)=\frac{\int_{A}\frac{P(p)}{P_{\text{max}}\left(p\right)e}P_{\text{max}}\left(p\right)\;dA}{\int_{A}P_{\text{max}}\left(p\right)\;dA}=\frac{\int_{A}P\left(p\right)\;dA}{\int_{A}P_{\text{max}}\left(p\right)\;dA}. \end{array}$$

This quantity was approximated by sampling each point in a 20 km grid over the whole analysis area (yielding *n* = 10,551 points), and replacing the integral by a sum:

$$\begin{array}{c} \alpha \left(A\right)\approx \frac{\sum_{j=1}^{n}P\left(p_{j}\right)}{\sum_{j=1}^{n}P_{\text{max}}\left(p_{j}\right)}. \end{array}$$

The overall relative potential *α*(*A*) comes with a minor caveat. The model does not consider competition for substrates between adjacent biogas plants, so a strict interpretation of *α*(*A*) should be that it measures the relative substrate utilization for only one randomly located biogas plant in absence of competitors. To utilize all substrates, some plants would have to face additional costs not described in this model because the disk-shaped collection areas of this model cannot be combined to cover the whole map without overlaps. However, this error can be expected to be negligible, as the whole map can be covered by regular hexagons of the same size as our disks (i.e. with the same substrate amounts) resulting only in a marginal increase in average collection distance.

More importantly, the error in the measure *α*(*A*) is obviously worse if substrates have very different collection distances *r*_*i*_, because it leads to a sort of double counting. For example, if straw can be collected from within a 30 km radius and manure only within 10 km, several different biogas plants could be on safe distance from each other’s manure sources while all counting the same straw on some field as an available co-substrate. To account for the effect of such competition it would be necessary to describe the substrate redistribution in a more detailed manner, e.g., explicitly model the location of plants and their substrate collection areas. However, the added complexity of such a treatment is not warranted considering the limited completeness and spatial resolution otherwise used in the model. To minimize the inherent problems of the proposed measure *α*(*A*), we instead chose to set all collection radii equal, i.e. *r*_*i*_ = *r*.

## Results

### Substrate amounts

**Fig 2:**
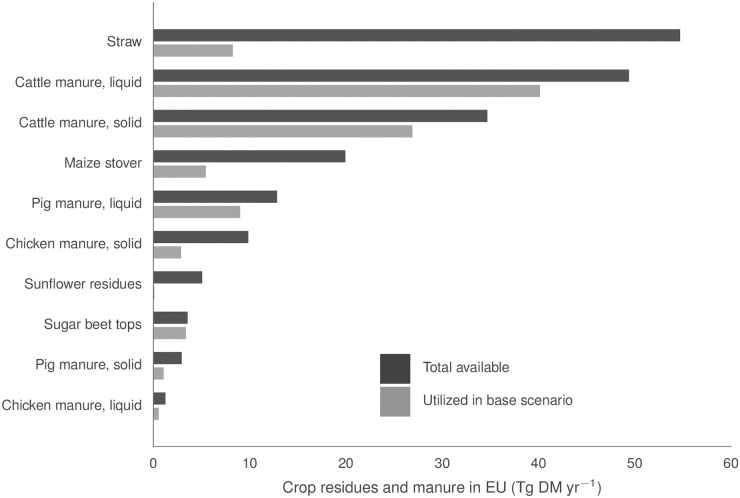
Available and utilized substrates in the base scenario. Black bars: Estimated available substrate quantities in EU28, excluding Greece, after accounting for the maximum removal rate from fields (40%, see Table 4) and the straw used for animal bedding. Straw used for bedding is excluded from the reported straw amounts and included in the solid manures. Manure fallen on pastures is not available as a substrate. Grey bars: Utilized substrates in the base scenario. 1 Tg DM = 1 million metric dry tonnes.

The estimated total available crop residues and manure in the base scenario, after accounting for maximal removal rate and other uses of crop residues, amounted to almost 200 million metric dry tonnes (Tg DM) per year, as shown in Fig 2. Roughly two thirds of the DM was found in straw (55 Tg DM yr^-1^), liquid cattle manure (49 Tg DM yr^-1^) and solid cattle manure (35 Tg DM yr^-1^, including straw bedding). Maize stover, liquid pig manure and solid chicken manure together contained another quarter of the DM, while all the other substrates accounted only for roughly 5% of the total.

**Fig 3:**
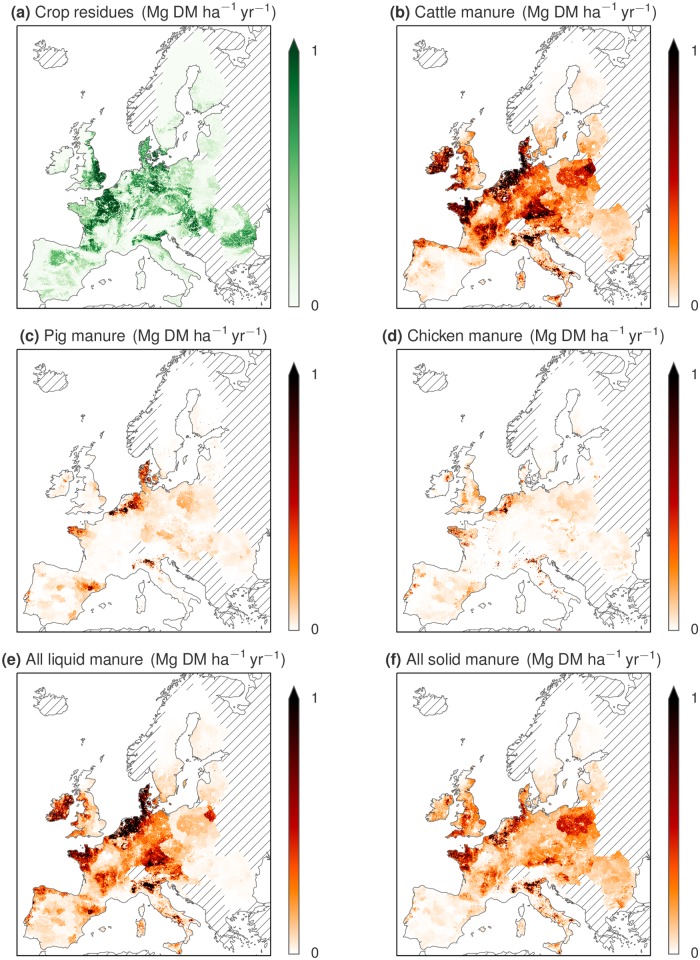
Estimated available crop residues and manure in the EU. The panels show estimated available amounts of (a) crop residues, (b) cattle manure, (c) pig manure, (d) chicken manure, (e) sum of liquid manure, and (f) sum of solid manure. The estimates account for the maximum sustainable removal of crop residues, and exclude manure that falls on pastures. Straw used for bedding is excluded from the crop residues density and included in the solid manure densities. Striped areas are not analyzed.

**Fig 4:**
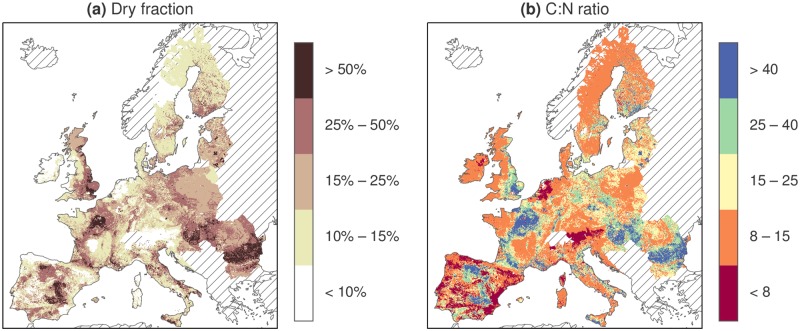
DM and C:N ratio of combined crop residues and manure. (a): DM concentration in the available substrates. (b): C:N ratio of the available substrates. Both maps concern the base scenario (see parameters in Table 4). C and N contents of substrates are given in Table 3. Striped areas are not analyzed.

However, the substrate composition varies widely across the EU, as seen in Fig 3. The maps illustrate some general patterns: First, crop residues are less common in mountainous areas. Second, liquid manure management is less common in eastern Europe, being almost nonexistent in some countries. Third, manure and crop residue concentrations are rather segregated in some areas, for example in Ireland and the UK, Portugal and Spain, parts of France, Poland and Romania. Due to this spatial segregation of animal production and arable farming, the C:N ratio and the DM concentration of the available substrate mixture varies widely between regions (see Fig 4).

### The biogas potential and its spatial distribution

The technical and economic constraints previously described lead to very substantial limitations on the biogas production potential from the substrate resources identified above. In the base scenario, we found that about three quarters of the manure substrates and one fifth of the crop residues could be used. The utilized share of each substrate in the base scenario is shown in Fig 2. In terms of biogas production, the potential in the base scenario was almost 0.7 EJ/year (HHV), or around half the theoretical potential assuming all available substrates could be digested. For comparison, the current EU-wide biogas production is about 0.6 EJ/year, of which less than 0.4 EJ/year is from agricultural sources (the remainder coming from landfills and sewage sludge) [5]. In Germany, which accounts for nearly half of this biogas production, less than 15% of the biogas comes from manure, and a few percent or less from crop residues [52]. Although no similar statistics on agricultural substrates seems to be available for the whole EU, this still indicates that most of the estimated potential of 0.7 EJ/year is so far not exploited.

The fraction of substrates utilized in the simulations varied considerably between countries since they have different composition and distribution of substrates. For example, regions with large amounts of crop residues tend to have very dry substrate mixtures and can therefore not utilize much of them if constrained to using wet digestion technology and assuming there are no additional, wetter co-substrates such as household waste or energy crops. In some regions (e.g. Denmark, the Netherlands, Belgium, and parts of Germany, France and Italy) it seems possible to utilize almost all substrates, while in others (e.g. Poland, Hungary, Romania, Finland, parts of UK and France) only a small fraction could be used. The maps showing substrate densities (Fig 3), C:N ratios and DM concentrations (Fig 4) help to illustrate the causes of this variation. Note, for example, that parts of France, Romania and Bulgaria have large amounts of available crop residues, leading to high DM concentrations and C:N ratios.

### Analysis of sensitivity to major parameter changes

To explore how the biogas potential depends on the economic and technical constraints discussed above, we formulated alternative scenarios by varying different combinations of two parameters at a time, in rather wide ranges of values. We express the results of these scenarios as the total biogas potential relative to the biogas potential in the base scenario, thereby focusing attention on the relative response to changes in parameters rather than on absolute numbers. Here, we present results from a selection of the alternative scenarios which exhibited large differences compared to the base scenario. Interpretations of these results are further discussed in the Discussion section below. Figures illustrating the full range of analyzed alternative scenarios are included in the S1 Appendix.

**Fig 5:**
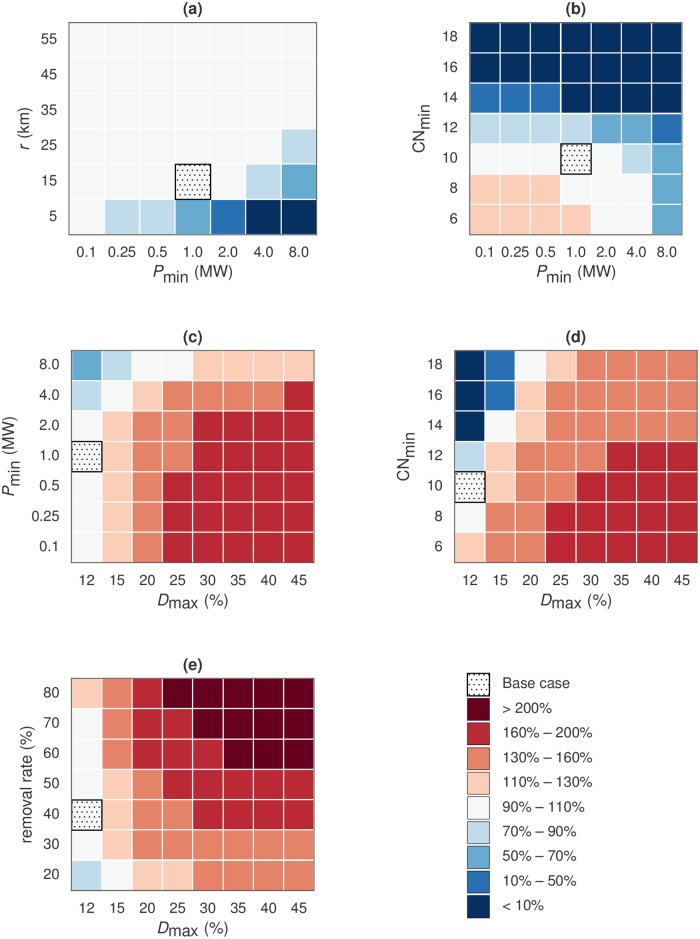
Simulated biogas potential when changing parameter values compared to the base scenario. The potential is expressed as a fraction of the base scenario potential. Each cell in each grid represents a different scenario, with all parameters as in the base scenario except for the two named parameters.

We first turn to the spatial density of substrates. Fig 5a shows that allowing smaller production units or longer collection distances than in the base scenario does not change the potential much. Hence, low substrate density alone is not a primary limitation in the base scenario. In other words, most manure and crop residues seems to be located in areas where a 15 km collection radius is sufficient to supply a 1 MW HHV plant. Even with a minimum plant size typically used in vehicle fuel production (e.g., 8 MW HHV), the same potential as in the base case can be achieved by extending the maximum collection radius from 15 to 35 km.

We noted above that only around one quarter of the crop residues and three quarters of the manure are utilized in the base scenario. The single most important constraint explaining this is the maximum DM content of substrate mixtures. As seen in Fig 5c–5e, the estimated potential increases by 60–100% if substrate mixtures with up to 30% DM content can be digested. This is primarily due to increased use of the dry crop residues (straw, stover, sunflower residues).

Relaxing the maximum DM constraint also affects the sensitivity of the estimated potential to other model constraints. For example, Fig 5e shows how the potential depends on the maximum removal rate for different maximum DM constraints. The maximum removal rate has a strong effect on the potential when *D*_max_ = 30%, which means that the potential is limited by the supply of crop residues if dry substrate mixtures can be handled. Similarly, Fig 5c shows that for *D*_max_ = 30%, a substantial amount of gas can be produced even when requiring relatively large plants, say 4–8 MW HHV.

**Fig 6:**
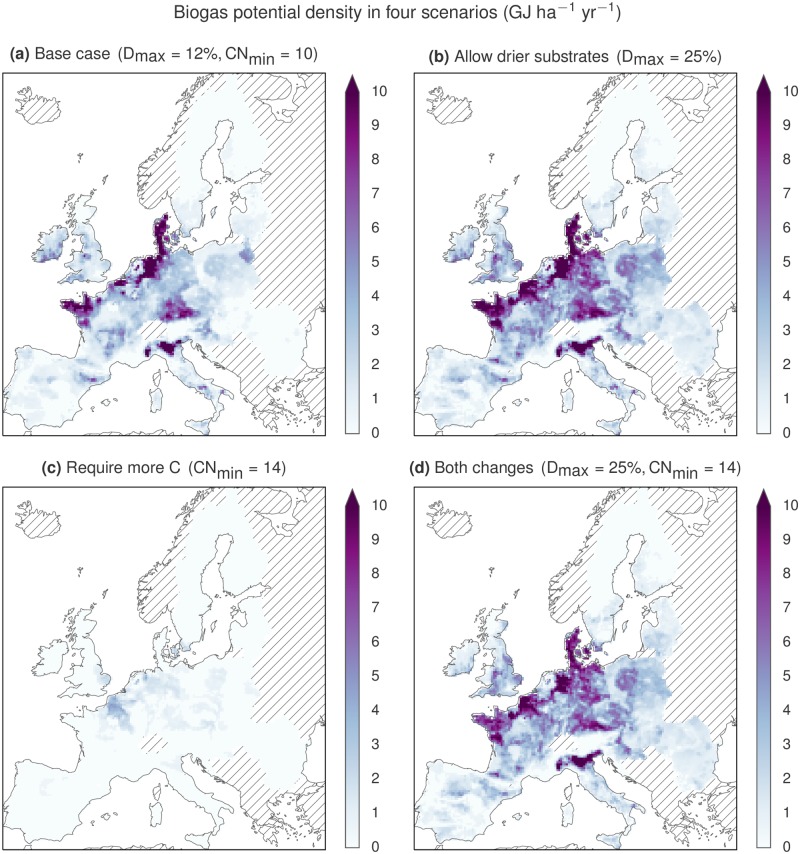
Biogas potential density in four different scenarios. Panel (a) shows the base case with production 0.7 EJ/year (see parameters in Table 4). Panel (b) shows that the potential is substantially increased (to 1.1 EJ/year) if drier substrate mixtures are allowed. Panel (c) shows that increasing the minimal C:N ratio of the substrate mixture from 10 to 14 severely diminishes the potential (to 0.1 EJ/year), all other things equal. However, as shown in panel (d), the extra carbon can be supplied (by crop residues) if 25% DM content is allowed, and in this case the potential is 0.9 EJ/year.

Another notable limitation is the minimum C:N ratio of the substrate mixture. Fig 5b shows that the simulated biogas production drops by more than 90% from the base scenario potential if the minimal C:N ratio is increased from 10 (base scenario) to 14 or above. This is because the nitrogen-rich manure substrates cannot be supplemented with carbon-rich crop residues in any large amounts without raising the DM content above the 12% allowed in the base scenario. Therefore, it makes sense to consider simultaneous modification of the minimum C:N ratio and the maximum DM content, as in Fig 5d. The figure illustrates the nonlinear effect these two constraint parameters have on the biogas potential: Relaxing the maximum DM constraint always has a strong positive effect on the simulated biogas production, but the relative effect is particularly large under stricter constraints on the minimum C:N ratio. The combined effect of these two constraints is also illustrated with maps in Fig 6.

Given these results it is not surprising, as shown in Fig 5e, that it makes almost no difference to change only the maximum removal rate of crop residues in the base scenario. This further illustrates that the base scenario is not primarily limited by access to crop residues. However, if a higher maximum DM content is allowed in substrate mixtures, the maximum removal rate has a large effect on the biogas production potential. In some of our most extreme scenarios, e.g. with 60% crop residue removal and up to 35% DM content, the production potential more than doubles compared to the base scenario.

## Discussion

### Relation to previous work

In this paper, we present a new method for assessing the potential for biogas production from manure and crop residues. To our knowledge, it is the first spatially explicit biogas potential model applied to the whole EU (except Greece, where land cover data is missing). The detail level of this model is somewhere in the middle between the least detailed studies, reporting total substrate amounts in a country or region, and the most detailed studies which, in addition to many different substrate sources, explicitly account for road networks, gas grids, investment costs, etc.

The model can be used to study the effects on the biogas potential of various technical and economic constraints. For example, we have shown that the biogas potential can be very different depending on the stringency of the constraints on minimum C:N ratio and maximum DM concentration of the substrate mixtures. Furthermore, we have shown that these two constraints are interrelated: Allowing a higher DM concentration always increases the biogas potential, but the relative effect is particularly large under stricter constraints on the minimum C:N ratio. This proves the general point that the biogas potential depends nonlinearly on the constraint parameters, or in other words, that the effect of changing two parameters simultaneously can be very different from the sum of effects from changing the parameters one at a time.

### Suggestions for future research

There is a considerable gap between the current biogas production from crop residues and manure and the potential estimated in this paper. A detailed analysis of the causes for this gap would be valuable if the goal is to understand how more of these substrates could be utilized. This question can partly be resolved both by complementing and comparing our work with other methods and by extending the model presented in this paper.

There are many potential limitations to the biogas production from crop residues and manure which we have not been able to cover in this paper, some of which may be hard to incorporate into our model. The profitability of biogas production depends, for example, on the cost of capital, energy prices, available subsidies and other support, the opportunity cost of the investment, and on the availability of equipment and expertise for digesting substrate mixtures rich in crop residues and manure. Furthermore, the inherent uncertainty in these factors is an economic risk that should be considered by potential investors. In principle, it is possible to include these factors in our model by making a more explicit economic model, but doing so would require significant effort since all the above-mentioned factors vary widely among EU member states.

Other issues, however, do seem more straightforward to address by refining our method. We believe that it would be useful to improve data quality and the model’s detail level where there is large uncertainty in data sources, where our results show a strong sensitivity to parameter changes, and where we know that important omissions have been made. Specifically, we identify four areas where better data or model extensions would be valuable.

First, it would make sense to add other substrates to the model, for example dedicated energy crops and suitable industrial or municipal waste. Doing so would have multiple effects on the model results since additional substrate streams would reduce overall transportation needs, increase the feasible production scale, and not least, change the chemical composition of available substrates (e.g., the overall DM content or C:N ratio). In this way, adding more substrates to the model may lead to increased utilization of the substrates already included, most notably the dry crop residues.

Second, since the biogas potential is rather sensitive to the maximum DM constraint, it could be useful to refine the model representation of DM constraints, perhaps by explicitly modeling both wet and dry digestion technology. Such a process model could include options for dilution or recirculation in wet digestion, pretreatment of lignocellulosic substrates, and development of better digester microbes.

Third, since results are also sensitive to the minimum C:N ratio constraint, we believe that a more detailed representation of the complex biochemical and technical issues related to chemical composition can lead to more accurate potential estimates.

Fourth, data on manure excretion and management systems in EU are scarce, incomplete and inconsistent. Since the utilization of crop residues is also limited by access to wet manure substrates, estimates could likely be changed substantially with improved manure data.

## Conclusion

We have presented a new approach to estimation of the EU-wide potential for sustainable biogas production from crop residues, manure, and, given relevant data, also from other substrates. Our results indicate that the main limitation to biogas production from crop residues is neither the actual availability of residues, nor their maximum sustainable removal from cropland or their transportation to biogas plants. Hence, refinements to these parts of our model would likely be of limited value. Rather, to make more accurate estimates of the potential with this model, there is a need for more data and analysis on manure excretion and management, the spatial distribution and costs for potential co-substrates, and the technical and biochemical constraints to digestion of substrate mixtures rich in crop residues.

## Supporting Information

S1 Appendix Further details on method and results. This PDF file contains some details on data sources, e.g. the exact Eurostat tables and National Inventory Reports to the UNFCCC that were used, and how we mapped different statistical nomenclatures to each other. It also contains results from 21 two-parameter sweeps, most of which are not included in the main text. The file also contains a link to a web site (http://rasmuse.github.io/biogas-residues-manure), containing (1) full source code needed to compute the results, (2) links to data sources, and (3) some interactive visualizations of results.
(PDF)

## References

[1] Persson UM. The impact of biofuel demand on agricultural commodity prices: a systematic review. Wiley Interdisciplinary Reviews: Energy and Environment. 2015;4(5):410–428. 10.1002/wene.155

[2] Hertel TW, Tyner WE. Market-mediated environmental impacts of biofuels. Global Food Security. 2013;2(2):131–137. 10.1016/j.gfs.2013.05.003

[3] European Commission. COM (2012) 595 final; 2012.

[4] European Parliament and Council. Directive 2015/1513 of the European Parliament and of the Council amending Directive 98/70/EC relating to the quality of petrol and diesel fuels and amending Directive 2009/28/EC on the promotion of the use of energy from renewable sources. Official Journal of the European Union. 2015;L(239).

[5] EurObserv’ER. Biogas barometer. EurObserv’ER; 2014.

[6] European Commission. State of play on the sustainability of solid and gaseous biomass used for electricity, heating and cooling in the EU SWD (2014) 259 final. Brussels: European Commission; 2014.

[7] Bentsen NS, Felby C. Biomass for energy in the European Union—a review of bioenergy resource assessments. Biotechnology for Biofuels. 2012;5(1):1–10. 10.1186/1754-6834-5-25 22546368 PMC3458922

[8] Dagnall S, Hill J, Pegg D. Resource mapping and analysis of farm livestock manures—assessing the opportunities for biomass-to-energy schemes. Bioresource Technology. 2000;71(3):225–234. 10.1016/S0960-8524(99)00076-0

[9] Zubaryeva A, Zaccarelli N, Del Giudice C, Zurlini G. Spatially explicit assessment of local biomass availability for distributed biogas production via anaerobic co-digestion—Mediterranean case study. Renewable Energy. 2012;39(1):261–270. 10.1016/j.renene.2011.08.021

[10] Perpiña C, Martínez-Llario JC, Pérez-Navarro A. Multicriteria assessment in GIS environments for siting biomass plants. Land Use Policy. 2013;31:326–335. 10.1016/j.landusepol.2012.07.014

[11] Höhn J, Lehtonen E, Rasi S, Rintala J. A Geographical Information System (GIS) based methodology for determination of potential biomasses and sites for biogas plants in southern Finland. Applied Energy. 2014;113:1–10. 10.1016/j.apenergy.2013.07.005

[12] Batzias FA, Sidiras DK, Spyrou EK. Evaluating livestock manures for biogas production: a GIS based method. Renewable Energy. 2005;30(8):1161–1176. 10.1016/j.renene.2004.10.001

[13] Bidart C, Fröhling M, Schultmann F. Livestock manure and crop residue for energy generation: Macro-assessment at a national scale. Renewable and Sustainable Energy Reviews. 2014;38:537–550. 10.1016/j.rser.2014.06.005

[14] Monforti F, Bódis K, Scarlat N, Dallemand JF. The possible contribution of agricultural crop residues to renewable energy targets in Europe: A spatially explicit study. Renewable and Sustainable Energy Reviews. 2013;19:666–677. 10.1016/j.rser.2012.11.060

[15] EEA. CLC2006 technical guidelines. European Environment Agency; 2007.

[16] Robinson TP, Wint GRW, Conchedda G, Van Boeckel TP, Ercoli V, Palamara E, et al Mapping the Global Distribution of Livestock. PLoS ONE. 2014;9(5):e96084 10.1371/journal.pone.0096084 24875496 PMC4038494

[17] Scarlat N, Martinov M, Dallemand JF. Assessment of the availability of agricultural crop residues in the European Union: Potential and limitations for bioenergy use. Waste Management. 2010;30(10):1889–1897. 10.1016/j.wasman.2010.04.016 20494567

[18] Nilsson D, Bernesson S. Halm som bränsle. Uppsala: Swedish University of Agricultural Sciences SLU; 2009 011.

[19] Kreuger E, Prade T, Björnsson L, Lantz M, Bohn I, Svensson SE, et al Biogas från skånsk betblast—potential, teknik och ekonomi Miljö- och Energisystem, Lunds Universitet; 2014 93.

[20] Monforti F, Lugato E, Motola V, Bodis K, Scarlat N, Dallemand JF. Optimal energy use of agricultural crop residues preserving soil organic carbon stocks in Europe. Renewable and Sustainable Energy Reviews. 2015;44:519–529. 10.1016/j.rser.2014.12.033

[21] Lugato E, Panagos P, Bampa F, Jones A, Montanarella L. A new baseline of organic carbon stock in European agricultural soils using a modelling approach. Global Change Biology. 2014;20(1):313–326. 10.1111/gcb.12292 23765562

[22] Vu VTK, Prapaspongsa T, Poulsen HD, Jørgensen H. Prediction of manure nitrogen and carbon output from grower-finisher pigs. Animal Feed Science and Technology. 2009;151(1-2):97–110. 10.1016/j.anifeedsci.2008.10.008

[23] NRC. Nutrient requirements of dairy cattle. 7th ed Washington D.C: National Academy Press; 2001.

[24] Leip A, Weiss F, Wassenaar T, Perez I, Fellman T, Loudjani P, et al. Evaluation of the Livestock Sector’s Contribution to the EU Greenhouse Gas Emissions (GGELS). Joint Research Centre, European Commission; 2010.

[25] Menzi H, Pain B, Smith K. Solid Manure in Europe. Results of a survey by the Working group on solid manure of RAMIRAN. RAMIRAN; 1998.

[26] Menzi H. Manure management in Europe: results of a recent survey. In: Proceedings of the 10th International Conference of the RAMIRAN Network. Štrbské Pleso, High Tatras, Slovak Republic: University of Veterinary Medicine, Slovak Republic; 2002.

[27] IPCC. 2006 IPCC Guidelines for National Greenhouse Gas Inventories. Eggleston S, Buendia L, Miwa K, Ngara T, Tanabe K, editors. IGES, Japan; 2006.

[28] Litorell O. Fjäderfägödsel—en värdefull resurs. Jordbruksverket; 2005. Jordbruksinformation 13.

[29] Sommer SG, Hutchings NJ. Ammonia emission from field applied manure and its reduction—invited paper. European Journal of Agronomy. 2001;15(1):1–15. 10.1016/S1161-0301(01)00112-5

[30] de Vries JW, Groenestein CM, de Boer IJM. Environmental consequences of processing manure to produce mineral fertilizer and bio-energy. Journal of Environmental Management. 2012;102:173–183. 10.1016/j.jenvman.2012.02.032 22459014

[31] Hamelin L, Naroznova I, Wenzel H. Environmental consequences of different carbon alternatives for increased manure-based biogas. Applied Energy. 2014;114:774–782. 10.1016/j.apenergy.2013.09.033

[32] Koch P, Salou T. AGRIBALYSE^®^: Rapport Méthodologique—Version 1.2 Angers. France; 2015.

[33] Møller HB, Sommer SG, Ahring BK. Methane productivity of manure, straw and solid fractions of manure. Biomass and Bioenergy. 2004;26(5):485–495. 10.1016/j.biombioe.2003.08.008

[34] Nahm Kh. Evaluation of the nitrogen content in poultry manure. World’s Poultry Science Journal. 2003;59(01):77–88. 10.1079/WPS20030004

[35] Miranda ND, Granell R, Tuomisto HL, McCulloch MD. Meta-analysis of methane yields from anaerobic digestion of dairy cattle manure. Biomass and Bioenergy. 2016;86:65–75. 10.1016/j.biombioe.2016.01.012

[36] Wang X, Lu X, Li F, Yang G. Effects of Temperature and Carbon-Nitrogen (C/N) Ratio on the Performance of Anaerobic Co-Digestion of Dairy Manure, Chicken Manure and Rice Straw: Focusing on Ammonia Inhibition. PLoS ONE. 2014;9(5):e97265 10.1371/journal.pone.0097265 24817003 PMC4016299

[37] Petersson A, Thomsen MH, Hauggaard-Nielsen H, Thomsen AB. Potential bioethanol and biogas production using lignocellulosic biomass from winter rye, oilseed rape and faba bean. Biomass and Bioenergy. 2007;31(11–12):812–819. 10.1016/j.biombioe.2007.06.001

[38] Bauer A, Leonhartsberger C, Bösch P, Amon B, Friedl A, Amon T. Analysis of methane yields from energy crops and agricultural by-products and estimation of energy potential from sustainable crop rotation systems in EU-27. Clean Technologies and Environmental Policy. 2009;12(2):153–161. 10.1007/s10098-009-0236-1

[39] Bossard M, Feranec J, Otahel J. CORINE land cover technical guide: Addendum 2000. Report 40. European Environment Agency; 2000.

[40] Walla C, Schneeberger W. The optimal size for biogas plants. Biomass and Bioenergy. 2008;32(6):551–557. 10.1016/j.biombioe.2007.11.009

[41] Lantz M. The economic performance of combined heat and power from biogas produced from manure in Sweden—A comparison of different CHP technologies. Applied Energy. 2012;98:502–511. 10.1016/j.apenergy.2012.04.015

[42] Bauer F, Persson T, Hulteberg C, Tamm D. Biogas upgrading—technology overview, comparison and perspectives for the future. Biofuels, Bioproducts and Biorefining. 2013;7(5):499–511. 10.1002/bbb.1423

[43] Petersson A, Wellinger A. Biogas upgrading technologies—developments and innovations. IEA Bioenergy Task 37; 2009.

[44] Berglund M, Börjesson P. Assessment of energy performance in the life-cycle of biogas production. Biomass and Bioenergy. 2006;30(3):254–266. 10.1016/j.biombioe.2005.11.011

[45] Weiland P. Biogas production: current state and perspectives. Applied Microbiology and Biotechnology. 2010;85(4):849–860. 10.1007/s00253-009-2246-7 19777226

[46] Nordberg A, Jarvis A, Stenberg B, Mathisen B, Svensson BH. Anaerobic Digestion of Alfalfa Silage with Recirculation of Process Liquid. Bioresource Technology. 2007;98(1):104–111. 10.1016/j.biortech.2005.11.027 16480862

[47] Estevez MM, Sapci Z, Linjordet R, Schnürer A, Morken J. Semi-Continuous Anaerobic Co-Digestion of Cow Manure and Steam-Exploded Salix with Recirculation of Liquid Digestate. Journal of Environmental Management. 2014;136:9–15. 10.1016/j.jenvman.2014.01.028 24534902

[48] Hamelin L, Wesnæs M, Wenzel H, Petersen BM. Environmental Consequences of Future Biogas Technologies Based on Separated Slurry. Environmental Science & Technology. 2011;45(13):5869–5877. 10.1021/es200273j 21671646

[49] Rajagopal R, Massé DI, Singh G. A critical review on inhibition of anaerobic digestion process by excess ammonia. Bioresource Technology. 2013;143:632–641. 10.1016/j.biortech.2013.06.030 23835276

[50] Shanmugam P, Horan NJ. Optimising the biogas production from leather fleshing waste by co-digestion with MSW. Bioresource Technology. 2009;100(18):4117–4120. 10.1016/j.biortech.2009.03.052 19395254

[51] Zheng Y, Zhao J, Xu F, Li Y. Pretreatment of lignocellulosic biomass for enhanced biogas production. Progress in Energy and Combustion Science. 2014;42:35–53. 10.1016/j.pecs.2014.01.001

[52] Scheftelowitz M, Rensberg N, Denysenko V, Daniel-Gromke J, Stinner W, Hillebrand K, et al Stromerzeugung aus Biomasse (Vorhaben Ila Biomasse) Zwischenbericht Mai 2015. DBFZ; 2015.

