# Supplementary material for: Analyzing key constraints to biogas production from crop residues and manure in the EU—A spatially explicit model
Source: PLoS One. 2017 Jan 31;12(1):e0171001. doi: 10.1371/journal.pone.0171001 (PMC5283681; doi:10.1371/journal.pone.0171001)
Supplement: S1 Appendix — This PDF file contains some details on data sources, e.g. the exact Eurostat tables and National Inventory Reports to the UNFCCC that were used, and how we mapped different statistical nomenclatures to each other. It also contains results from 21 two-parameter sweeps, most of which are not included in the main text. The file also contains a link to a web site (http://rasmuse.github.io/biogas-residues-manure), containing (1) full source code needed to compute the results, (2) links to data sources, and (3) some interactive visualizations of results. (PDF) [file pone.0171001.s001.pdf]

Supporting Information: The potential for biogas production from crop residues and manure in the EU, accounting for key technical and economic constraints

Rasmus Einarsson and U. Martin Persson

1 Source code and interactive visualizations

Source code and interactive visualizations of some results from the paper can be found online at: `https://rasmuse.github.io/biogas-residues-manure`

2 Crop residues

Data on harvests were collected from three different tables in Eurostat:

- agr_r_crops, listing harvested amounts of some of the investigated crops (wheat, barley, sugar beet, rapeseed and turnip rape, sunflower seed) for most NUTS2 regions (NUTS1 for Germany and the UK) in each year;
- ef_oluaareg, listing planted areas of all the investigated crops on NUTS2/NUTS1 level, as measured in the triannual farm structure survey (years 2007, 2010, 2013, etc);
- apro_cpp_crop, listing harvested amounts of all the investigated crops on NUTS0 (national) level in each year.

Where available, we used data from agr_r_crops, averaged over the years 2009–2011. When not available, we estimated the subnational harvest data by assuming a constant area yield in each country, i.e., distributing the total NUTS0 harvest (apro_cpp_crop data) proportional to the NUTS2/NUTS1 planted areas (ef_oluaareg data) for the year 2010.

The nomenclature in these table is not completely identical across tables. We connect crops to statistical codes as shown in Table 1.

Table 1 – Crops and Eurostat statistical codes.

Crop	Eurostat names	Codes in agr_r_crops and ef_oluaareg tables	Codes in apro_cpp_crop table
Wheat	Common wheat and spelt, durum wheat	C1120, C1130	B_1_1_1_HA, B_1_1_2_HA
Rye	Rye (Rye and maslin if rye not available)	C1150 (C1140)	B_1_1_3_HA
Barley	Barley	C1160	B_1_1_4_HA
Oats	Oats	C1180	B_1_1_5_HA
Grain maize	Grain maize	C1200 (C1201)	B_1_1_6_HA
Sugar beet	Sugar beet	C1370	B_1_4_HA
Rapeseed	Rape and turnip rape	C1420	B_1_6_4_HA
Sunflower seed	Sunflower seed	C1450	B_1_6_5_HA

3 Manure

3.1 Animal populations

Animal populations were taken from the triannual farm structure survey, Eurostat table `ef_olsaareg`. We used data from 2010.

The classification of animals used for manure management systems in the National Inventory Reports (NIRs) is more aggregated than the Eurostat population data, so we aggregated the animal populatios from `ef_olsaareg` into the NIR manure management classes as follows. We used the average manure management reported in the NIRs for years 2009–2011 (in the 2014 submissions).

- **Cattle, option A**
  - Dairy cattle: `C_2_6_HEADS`
  - Non-dairy cattle: `C_2_1_HEADS`, `C_2_2_HEADS`, `C_2_4_HEADS`, `C_2_3_HEADS`, `C_2_5_HEADS`, `C_2_99_HEADS`
- **Cattle, option B**
  - Mature dairy cattle: `C_2_6_HEADS`
  - Mature non-dairy cattle: `C_2_4_HEADS`, `C_2_99_HEADS`
  - Young cattle: `C_2_1_HEADS`, `C_2_2_HEADS`, `C_2_3_HEADS`, `C_2_5_HEADS`
- Breeding pigs: `C_4_2_HEADS`
- Fattening pigs: `C_4_99_HEADS`
- Laying hens: `C_5_2_1000_HEADS`
- Broilers: `C_5_1_1000_HEADS`

The two different options for cattle classification were both included because manure management systems are reported in the NIRs according to either one of them (chosen by the reporting country).

The population data was multiplied by the excretion factors (see Table 2) to obtain total excreted amounts per animal category and manure management class. Before downscaling the manure amounts spatially, we aggregated them to the GLW2 classes (cattle, pigs, chickens).

3.2 Manure management systems

In the NIRs, seven manure management systems are included, which we aggregated into three classes (liquid, solid, unavailable) according to Table 3.

Countries did not seem to use the National Inventory classifications completely in the same way. For example, some countries (e.g. Sweden and Denmark) seemed to use the category “Other” to indicate deep bedding (a.k.a. bedded pack) systems, while others (e.g. UK, Germany and France) appear to include such systems in the “Solid storage” category. In face of these ambiguities, we chose to only include one type of solid manure system.

Table 2 – Assumed excretion and total manure production per animal head for different animals and manure management systems. Excretion values for cattle and pigs from IPCC (2006). Excretion for poultry based on Litorell (2005).

Animal	Excretion (kg VS head^{-1} d^{-1})	System	Manure incl. bedding (kg VS head^{-1} d^{-1})
Dairy cows	5.1	liquid	5.1
		solid	10.2
Other cattle	2.6	liquid	2.6
		solid	5.2
Breeding pigs	0.5	liquid	0.5
		solid	1.0
Fattening pigs	0.3	liquid	0.3
		solid	0.6
Laying hens	$8 \cdot 10^{-3}$	liquid	$8 \cdot 10^{-3}$
		solid	$8 \cdot 10^{-3}$
Broilers	$5 \cdot 10^{-3}$	liquid	$5 \cdot 10^{-3}$
		solid	$5 \cdot 10^{-3}$

We also noted that Spain reports 100% of its manure management in the "Other" category. Instead of interpreting this as 100% solid manure, we assigned Portugal's distribution of manure management systems also to Spain.

The description of solid manure systems is especially problematic since there seems to be a very wide variation within this category. The word "solid manure" may be used to describe everything from relatively wet (around 15% DM) mixtures of manure and straw, removed daily, to deep bedding (roughly 25–30% DM) which is stored and partially composted for several months before removal. We have only encountered anecdotal evidence on the amounts and types of bedding material used in "solid" systems. Loosely based on such evidence we chose to assume that in solid manure systems for cattle and pigs, bedding straw was added to excretions in the ratio 1:1, counted in volatile solids basis. For chickens in solid manure systems, we assumed the bedding material was wood chips and that the wood chips did not add any volatile solids.

Table 3 – Aggregation of manure management systems in National Inventory Reports to the three classes used in our analysis.

Management system	National Inventory Report classification
Liquid	Anaerobic lagoon, Liquid system
Solid	Solid storage, Dry lot, Other
Unavailable	Pasture range paddock, Daily spread

4 Parameter sweeps

Results of parameter sweeps are shown below. Each cell in each grid shows the biogas potential relative to the base scenario, when taking the base scenario and changing two parameters as indicated.

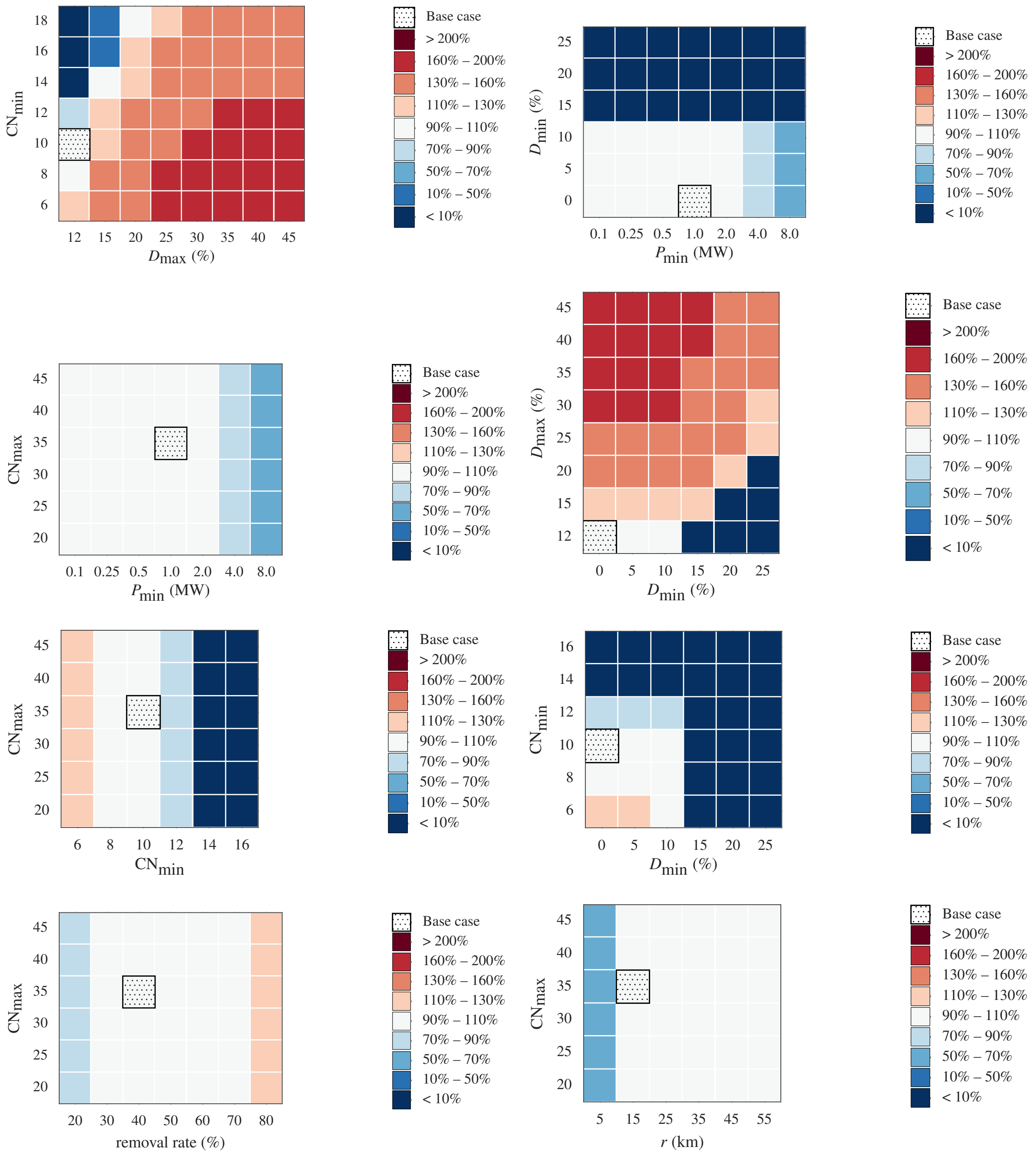

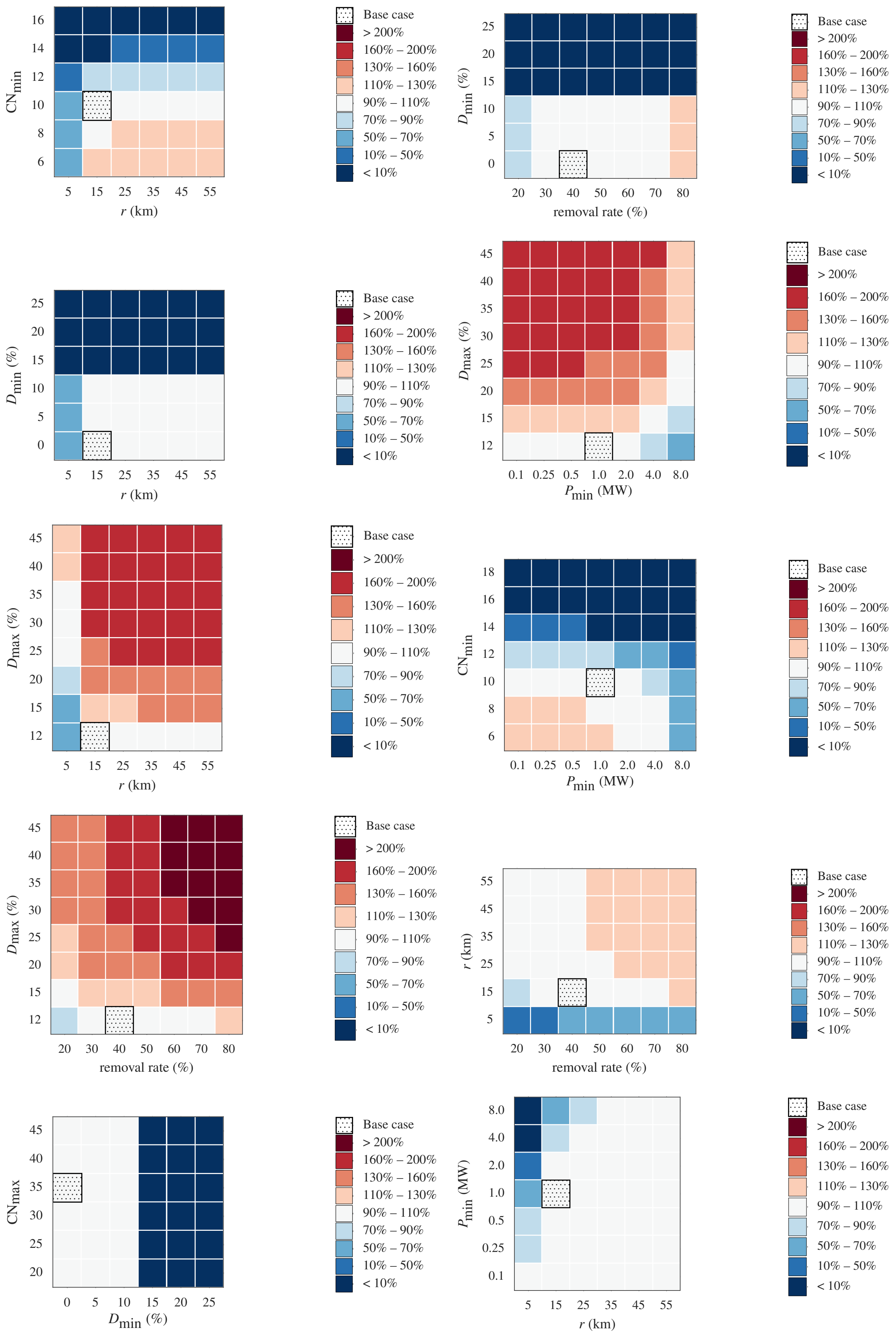

CNmin
16
14
12
10
8
6
5 15 25 35 45 55
r (km)
Base case
> 200%
160% – 200%
130% – 160%
110% – 130%
90% – 110%
70% – 90%
50% – 70%
10% – 50%
< 10%
Dmin (%)
25
20
15
10
5
0
20 30 40 50 60 70 80
removal rate (%)
Dmin (%)
5 15 25 35 45 55
r (km)
Dmax (%)
45
40
35
30
25
20
15
12
0.1 0.25 0.5 1.0 2.0 4.0 8.0
Pmin (MW)
Dmax (%)
5 15 25 35 45 55
r (km)
CNmin
18
16
14
12
10
8
6
0.1 0.25 0.5 1.0 2.0 4.0 8.0
Pmin (MW)
Dmax (%)
20 30 40 50 60 70 80
removal rate (%)
r (km)
55
45
35
25
15
5
20 30 40 50 60 70 80
removal rate (%)
CNmax
45
40
35
30
25
20
0 5 10 15 20 25
Dmin (%)
Pmin (MW)
8.0
4.0
2.0
1.0
0.5
0.25
0.1
5 15 25 35 45 55
r (km)

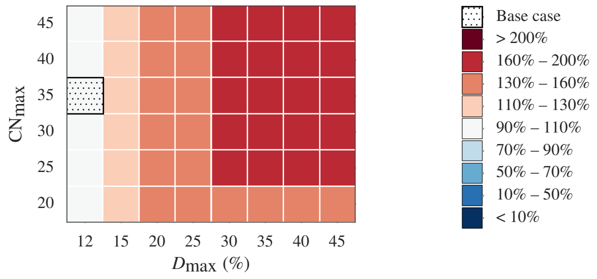
CNmax
45
40
35
30
25
20
12 15 20 25 30 35 40 45
Dmax (%)
Base case
> 200%
160% – 200%
130% – 160%
110% – 130%
90% – 110%
70% – 90%
50% – 70%
10% – 50%
< 10%

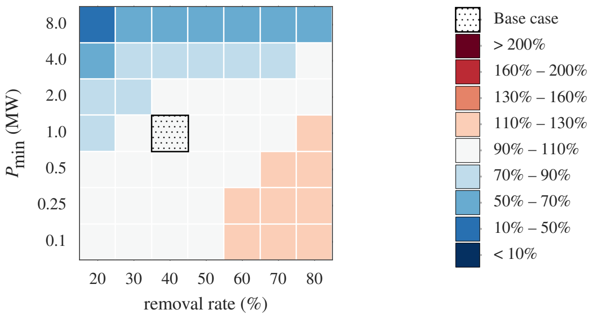
Pmin (MW)
8.0
4.0
2.0
1.0
0.5
0.25
0.1
20 30 40 50 60 70 80
removal rate (%)
Base case
> 200%
160% – 200%
130% – 160%
110% – 130%
90% – 110%
70% – 90%
50% – 70%
10% – 50%
< 10%

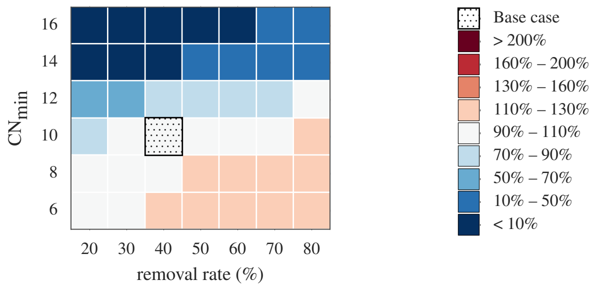
CNmin
16
14
12
10
8
6
20 30 40 50 60 70 80
removal rate (%)
Base case
> 200%
160% – 200%
130% – 160%
110% – 130%
90% – 110%
70% – 90%
50% – 70%
10% – 50%
< 10%

References

IPCC (2006) *2006 IPCC Guidelines for National Greenhouse Gas Inventories*. Ed. by Simon Eggleston, Leandro Buendia, Kyoko Miwa, Todd Ngara, and Kiyoto Tanabe. IGES, Japan.

Litorell, Ove (2005) *Fjäderfägödsel - en värdefull resurs*. Jordbruksinformation 13. ISSN 1102-8025. Jordbruksverket.
